# Insertion of a TRIM-like sequence in *MdFLS2-1* promoter is associated with its allele-specific expression in response to *Alternaria alternata* in apple

**DOI:** 10.3389/fpls.2022.1090621

**Published:** 2022-12-29

**Authors:** Zhaolin Liang, Kai Liu, Chunyang Jiang, An Yang, Jiadi Yan, Xiaolei Han, Caixia Zhang, Peihua Cong, Liyi Zhang

**Affiliations:** ^1^ Research Institute of Pomology, Chinese Academy of Agricultural Sciences, Xingcheng, China; ^2^ Key Laboratory of Biology and Genetic Improvement of Horticultural Crops (Germplasm Resources Utilization), Research Institute of Pomology, Chinese Academy of Agricultural Sciences, Ministry of Agriculture, Xingcheng, China

**Keywords:** *alternaria alternata* apple pathotype, *malus domestica* borkh., allele-specific expression, MdFLS2, TRIM element

## Abstract

Alternaria blotch disease, caused by *Alternaria alternata* apple pathotype (AAAP), is one of the major fungal diseases in apple. Early field observations revealed, the anther-derived homozygote Hanfu line (HFTH1) was highly susceptible to AAAP, whereas Hanfu (HF) exhibited resistance to AAAP. To understand the molecular mechanisms underlying the difference in sensitivity of HF and HFTH1 to AAAP, we performed allele-specific expression (ASE) analysis and comparative transcriptomic analysis before and after AAAP inoculation. We reported an important immune gene, namely, *MdFLS2*, which displayed strong ASE in HF with much lower expression levels of HFTH1-derived alleles. Transient overexpression of the dominant allele of *MdFLS2-1* from HF in GL-3 apple leaves could enhance resistance to AAAP and induce expression of genes related to salicylic acid pathway. In addition, *MdFLS2-1* was identified with an insertion of an 85-bp terminal-repeat retrotransposon in miniature (TRIM) element-like sequence in the upstream region of the nonreference allele. In contrast, only one terminal direct repeat (TDR) from TRIM-like sequence was present in the upstream region of the HFTH1-derived allele *MdFLS2-2.* Furthermore, the results of luciferase and β-glucuronidase reporter assays demonstrated that the intact TRIM-like sequence has enhancer activity. This suggested that insertion of the TRIM-like sequence regulates the expression level of the allele of *MdFLS2*, in turn, affecting the sensitivity of HF and HFTH1 to AAAP.

## 1 Introduction

Apple (*Malus domestica* Borkh.) is one of the significant fruit species that is widely consumed globally. However, apple is susceptible to fungal diseases during its cultivation. Alternaria blotch disease, caused by *Alternaria alternata* apple pathotype (AAAP), is one of the most serious fungal diseases of apples (Filajdic and Sutton, 1991; Zhang et al., 2015). As a pathogenic variant of *Alternaria*, AAAP mainly infects apple leaves and causes early defoliation of the tree, severely damaging the yield and quality of apples (Moriya et al., 2019). The most effective measure for overcoming fungal diseases in apple is the selective breeding of resistant cultivars (Zhang et al., 2018). However, the mechanism of resistance to AAAP in apples is still not clearly studied (Hou et al., 2021). Therefore, it is urgent to investigate the molecular mechanism underlying Alternaria blotch disease resistance in apple.

To defend themselves against pathogens, plants have evolved with strong immune mechanisms (Ngou et al., 2022). Pattern recognition receptors (PRRs) located on plant cell membranes recognize conserved molecular pathogen-molecular patterns (PAMPs) secreted by pathogens, thereby activating pattern-triggered immunity (PTI) to limit pathogenicity (Zipfel, 2008). In contrast, nucleotide-binding leucine-rich repeat receptors located inside plant cells can activate effector-triggered immune (ETI) responses by directly or indirectly sensing specific effectors secreted by pathogens (Nomura et al., 2011). PTI is an important component of basal resistance in plants. Recent studies have reported that defense responses triggered by PTI are equally essential for ETI activation (Ngou et al., 2021; Yuan et al., 2021). As PRRs trigger PTI responses, they have been at the forefront of plant immune research, with the receptor kinase *FLS2* being the most extensively studied (Zipfel et al., 2004; Lu et al., 2011; Shi et al., 2016; Zhang et al., 2020b). FLS2 can form a complex with the co-receptor kinase BAK1 to recognize the conserved protein polypeptide flg22 in the flagellin of pathogenic bacteria to activate the immune response of plants (Sun et al., 2013). In addition, FLS2 may be involved in the recognition of other ligands, e.g., the sensitivity of *Arabidopsis AtFLS2* mutants to the protein polypeptide *Ax21* secreted by *Xanthomonas oryzae* pv. *oryzae* (Xoo) is significantly reduced by the action of FLS2 (Danna et al., 2011). PTI-mediated immune responses involve MAPK cascade responses and phytohormone signaling (Asai et al., 2002; Kong et al., 2016). Upon recognition of flg22 by FLS2, the MAPK cascade response in plants is activated to further phosphorylate WRKY transcription factors, thereby regulating the expression of immune-related genes (Sarowar et al., 2019). Previous studies in apple reported one *FLS2* ortholog, namely, *MdFLS2* (Chen-Hui et al., 2018). Overexpression of *MdFLS2* in *Arabidopsis* increased its resistance to *Botryosphaeria dothidea*, whereas the expression levels of genes related to salicylic acid pathway were significantly enhanced (Liu et al., 2018). However, it is unclear whether *MdFLS2* has a broad-spectrum resistance to various plant pathogens, similar to *AtFLS2*, and the mechanisms regulating its expression level should be further studied.

**Figure 1:**
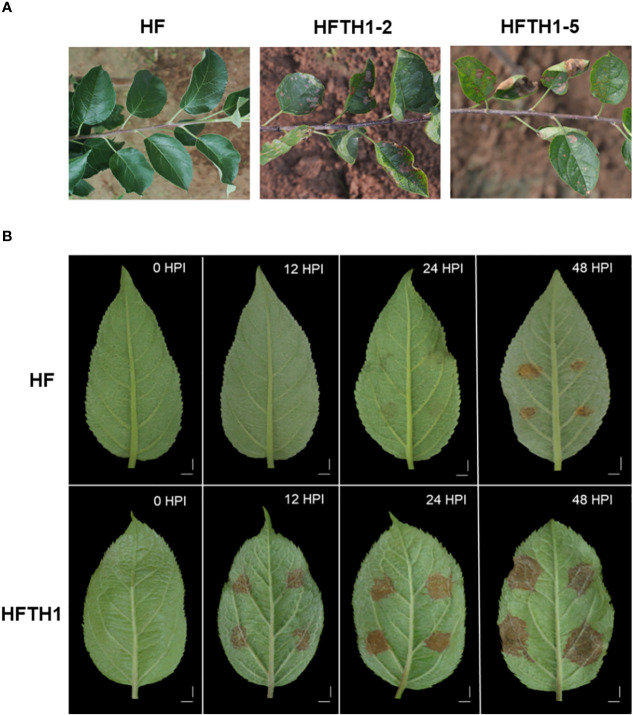
Different susceptibility of HF and HFTH1 to AAAP. **(A)** Investigation of the susceptibility of HF and HFTH1 to AAAP in the field experiments. **(B)** Changes in symptoms of HF and HFTH1 at 12, 24, and 48 h after inoculation with AAAP *in vitro*. Mock inoculation of HF and HFTH1 leaves without AAAP was used as the control (0HPI). Scale bar = 1 cm.

Apple is typically hetero-pollinated plant, and its highly heterozygous genotypes confer diversity in response to biotic (Reim et al., 2019) and abiotic (Ren et al., 2017) stresses. After 3 years of field observations, we observed that Hanfu (HF) exhibited resistance, whereas the anther-derived homozygote Hanfu line (HFTH1) was highly susceptible to AAAP (**Figure 1A**). The homozygosis of some important genes predisposes plants to exhibit extreme phenotypes (Wang et al., 2014; Zhang et al., 2020a). However, heterozygotes can overcome these adverse effects by selecting more favorable allele expression under specific conditions, i.e., allele-specific expression (ASE) (Shao et al., 2019). Recent studies have reported that ASE is involved in the regulation of many important traits in apples, such as fruit ripening (Sun et al., 2020) and flower color formation (Tian et al., 2022). To understand the molecular mechanisms underlying the difference in sensitivity of HF and HFTH1 to AAAP, we aimed to investigate the ASE patterns in HF under AAAP stress. Previously, we have obtained the HFTH1 haplotype reference genome (Zhang et al., 2019), providing a genomic resource for ASE analysis in HF. In this study, we integrated ASE analysis and comparative transcriptomic analysis of HF and HFTH1 before and after AAAP inoculation to identify key genes and regulatory patterns that influence differences in susceptibility to AAAP. These results can provide new insights and references for breeding with Alternaria blotch disease resistance in apple.

## 2 Materials and methods

### 2.1 Plant materials, AAAP culture, and leaf inoculation

To reduce the genetic difference between two apple species, we selected HF (exhibiting resistance to AAAP) and HFTH1 (an anther-derived homozygote Hanfu line susceptible to AAAP) as experimental materials. Both materials were grafted on GM256 rootstock in 2010 and grown in a research orchard at Research Institute of Pomology, Chinese Academy of Agricultural Sciences (CAAS). AAAP was obtained from Plant Protection Center of Institute of Pomology, CAAS. AAAP was cultured on potato dextrose agar (PDA; 200 g potato extract, 20 g dextrose, and 20 g agar in 1 L water) medium and incubated in dark until the mycelia spread to two-third of the plate. The mycelium was inoculated onto 20-day-old HF and HFTH1 leaves according to the experimental method described previously (Zhu et al., 2017). In each treatment, four cakes of mycelium were used to inoculate both sides of the midvein on the abaxial surface of the leaves. Six treatment groups were set up at 12-h intervals for both plant materials, and the control group was mock-inoculated using PDA medium cakes without mycelium. After inoculation, the apple leaves were incubated in a sterilized culture chamber at 25°C under a 14 h light/10 h dark conditions with moisture control. Further, 48 h after inoculation in the first group, samples were simultaneously collected from each group to observe the incidence of the two materials at three stages after inoculation (12 HPI, 24 HPI, and 48 HPI) compared with the control group (0 HPI). Apple leaves were collected from three HF plants and used for whole genome resequencing.

Leaf samples were quickly placed in liquid nitrogen and stored at -80°C for resequencing. Three parallel leaves on three trees were collected for each sample set, representing three independent biological replicates. The strand-specific transcriptome sequencing and whole genome resequencing was performed by BerryGenomics (Beijing, China).

### 2.2 Genome and strand-specific transcriptome sequencing

Total cellular DNA was extracted using the DNeasy Plant Mini kit (Qiagen, Hilden, Germany), as per the manufacturer’s instructions. The final libraries constructed above were sequenced using the Illumina Novaseq6000 platform, and 150-bp paired-end reads were generated with an insert size of approximately 350 bp. The raw reads obtained from sequencing were quality controlled, and the clean reads obtained after screening were compared with the HFTH1 reference genome (Zhang et al., 2019) using BWA (version 0.7.15; Li and Durbin, 2009) for variant calling.

As per the manufacturer’s instructions, total RNA was isolated using cetyltrimethyl ammonium bromide method (Pavy et al., 2008). The purity of RNA was measured using NanoDrop 2000 spectrophotometer (Thermo Fisher Scientific, USA) and analyzed for RNA degradation and contamination using agarose gel electrophoresis. Strand-specific cDNA libraries were constructed using the dUTP method, as per a previous study (Parkhomchuk et al., 2009). Further, the 24 individual libraries were sequenced using the Illumina Novaseq6000 platform (BerryGenomics, Beijing, China).

### 2.3 Analysis of data and allele−specific expression

The raw reads of sequenced raw sequences were subjected to strict quality control to obtain valid and high-quality clean reads. The clean reads were compared with the HFTH1 apple reference genome using HISAT2 (v 2.20; Kim et al., 2015) to obtain information such as the position of the read on the compared genome and quality of the match; thus, the gene or transcript is annotated and quantified. Gene-level quantification was performed using featureCounts (Version 2.0.1; Liao et al., 2014), and gene expression levels were calculated as FPKM. Based on the comparison results, the differential expression of genes in each sample was analyzed using edgeR package (Robinson et al., 2010), and the pvalue and padj values of differential expression were calculated. The differential genes with padj < 0.05 and |log2FoldChange| > 1 were screened. GO enrichment analysis was performed using topGO software, and KEGG enrichment analysis was performed using KOBAS 3.0 (http://kobas.cbi.pku.edu.cn/). The heatmap was created using Tbtools (Chen et al., 2020).

To identify alleles, genome and transcriptome sequencing data compared with the reference genome were called for variants using GATK (version 3.8.0; Brouard et al., 2019). In this study, only SNPs were used to distinguish alleles using VCFTools (version 0.1.14) to filter SNPs and retaining SNP sites with read depths above 8×, the remaining SNP sites were annotated using snpEff (Cingolani et al., 2012). The SNP sites located in the CDS region of the genome were used for the identification of ASE. Genes that simultaneously satisfy the following conditions were considered ASE genes (ASEGs; Tian et al., 2022): (1) read counts of the reference allele divided by total read counts were >0.75 or <0.25, (2) different SNPs on the same gene exhibited same direction of significant bias, and (3) genes were identified as ASEGs in at least two out of three replicates.

### 2.4 Vector construction and transient overexpression in apple leaves

Specific primers were designed to amplify *MdFLS2-1* and *MdFLS2-2* in HF based on the coding sequence of *MdFLS2* predicted from the HFTH1 reference genome (**Supplementary Table S7**). The CDS region of *MdFLS2* allele was inserted into the *Nde I* digest site of PRI101 vector using 2× MultiF Seamless Assembly Mix (ABclonal, Wuhan, China) according to the in-fusion cloning method. Specific primers were designed to amplify the 2058-bp promoter sequence of *MdFLS2* allele based on the HFTH1 reference genome (**Supplementary Table S7**) and ligated to the pESI-Blunt vector. The ligated vector was transformed into *E. coli* DH5α competent cells, and the monoclonal colonies were picked and sent to GENEWIZ (Tianjin, China) for sequencing. The allelic variants of the gene were analyzed using ClustalX 2.1.

A recombinant plasmid inserted with the CDS region of *MdFLS2-1* was transformed into *Agrobacterium tumefaciens* strain GV3101 (Bai et al., 2013). The vector-transformed *Agrobacterium* was cultured in 10 mL YEP medium and shaken at 28°C and 130 rpm until OD_600_ reached to 1. The bacterial broth was centrifuged at 5000 rpm, resuspended in 10 mL of buffer (10 mM MgCl_2_,10 mM MES [pH 5.7], and 200 μM acetosyringone), and used after 3 h of activation at 28°C. To improve the transformation efficiency, we selected GL-3 apple plants that were favorable for transient transformation experiments. GL-3 apple plants cultured *in vitro* for 1 month were selected, and two spots on both sides of the leaf veins were selected to be infiltrated by injection. The infiltrated plants were placed in new MS medium and incubated for 4 days at 25°C in a light incubator under 16 h light/8 h dark conditions. Three *Agrobacterium*-infiltrated plants were randomly selected, and RNA from the injected leaves was extracted for gene expression analysis. After 4 days of infiltration, the remaining plants were inoculated with AAAP spore suspension at the location of the *Agrobacterium* injection, and the inoculated plants were placed back into MES medium and incubated in a light incubator for 48 h. In the control group, GL-3 apple leaves were treated in the same manner with sterile injection buffer and injection buffer containing empty vector, and the same batch of AAAP spore suspension was used to inoculate the infiltration site of the leaves. Plants with AAAP-infected leaves were examined at 48 HPI, and three independent biological replicates were collected and photographed. The differences in incidence levels were analyzed using student’s t test (Zhang et al., 2018).

### 2.5 Luciferase reporter assay

The TRIM-like sequence from the promoter of *MdFLS2-1* and TDR2 sequence from the promoter of *MdFLS2-2* were respectively fused to the cauliflower mosaic virus 35S minimal promoter (mpCaMV) and ligated to the *HindIII*-*BamHI* site of pGrernII0800-*LUC* vector, to generate the construct structures TRIM:mpCaMV : *LUC* and TDR2:mpCaMV : *LUC*. Sequences were synthesized (**Supplementary Table S8**) by GENEWIZ (Tianjin, China). Two reporter constructs were used to simultaneously transform *Agrobacterium* GV3101. Leaves of 8-week-old *Nicotiana benthamiana* plants were permeated with a needle-free syringe using the above injection buffer. The infiltrated plants were first incubated in a phytotron at 23°C for 12 h, followed by 60 h of growth under 16 h light/8 h dark conditions. The working solution (100 mM stock solution; 0.1% Triton X-100) was evenly spread onto the plant leaves using a high-pressure sprayer. The leaves were left in dark for 7 min, and the LUC images were captured using a low-light cooled CCD imaging apparatus (Tanon 5200Multi, China). The LUC/REN ratio was analyzed as described in a previous study (Liu et al., 2019). The experiment was independently repeated three times to obtain similar results.

### 2.6 Analysis of β-glucuronidase activity

The TRIM-like and TDR2 sequences fused with the 35S minimal promoter were respectively ligated to the *HindIII*-*BamHI* site of the pBI121 vector to generate reporter constructs and used to transform *Agrobacterium* GV3101. The GV3101 strain containing the recombinant plasmid was infiltrated into the abaxial leaf surface of *N. benthamiana* plants using the injection buffer described above. Three biological replicates were set for each infection. Infected plants were grown in a climatic chamber for 3 days, and β-glucuronidase (GUS) activity was detected as described in a previous study (Jefferson et al., 1987).

### 2.7 RNA extraction and RT-qPCR analysis

After freezing the plant leaves in liquid nitrogen, total RNA was extracted using Quick RNA Isolation Kit (Huayueyang Biotechnology, China) according to the manufacturer’s instructions. RNA quality was detected using agarose gel electrophoresis, and RNA concentration was determined using NanoDrop 2000 spectrophotometer (Thermo Fisher Scientific, USA). The same concentration of RNA was reverse transcribed to cDNA using PrimeScript RT Master Mix (TakaRa, Tokyo, Japan). RT-qPCR was conducted using a TB-Green PCR kit (TakaRa, Tokyo, Japan) as per the manufacturer’s instructions. The relative expression of each gene was calculated using the 2^-ΔΔCt^ method (Livak and Schmittgen, 2001) using *Tubulin* as the reference gene to normalize the gene expression level. Three experimental replicates were set up for each sample, and the mean standard deviation was calculated. RT-qPCR primers (**Supplementary Table S7**) were designed using primer-BLAST (https://www.ncbi.nlm.nih.gov/tools/primer-blast).

## 3 Results

### 3.1 HF and HFTH1 have different sensitivity to AAAP

Based on field observation for 3 years, the homozygous Hanfu line HFTH1 exhibited symptoms of severe spotted defoliation disease (**Figure 1A**), causing early defoliation in the wet rainy season. Meanwhile, the heterozygote HF exhibited no symptoms of susceptibility to the disease.

We performed simultaneous infection experiments on isolated leaves of HF and HFTH1 using the AAAP strain and recorded the symptoms on leaves (**Figure 1B**). The leaves of HFTH1 started exhibiting tissue necrosis by 12 HPI, brown spots by 24 HPI, and dark brown spots with conspicuous white hyphae on the necrotic tissue by 48 HPI. In contrast, HF leaves exhibited a later onset and milder symptoms. By 12HPI, no signs of disease were observed on HF leaves. By 24HPI, slight symptoms of the disease appeared. By 48HPI, lesions expanded and deepened to brown.

### 3.2 Sequencing and identification of ASE

The leaves of HF and HFTH1 after 12, 24, and 48 h of AAAP inoculation were set up in three replicates. We mixed the mock-inoculated leaves of three time points into one sample as the control (0 HPI). A total of 24 samples were sequenced using the Illumina Novaseq6000 platform for Strand-specific transcriptome sequencing. In total, 20,379,378–36,174,753 clean reads were generated from 24 RNA-seq libraries, with Clean GC% of 45.43–46.52, CleanQ20 of 97.33–98.10, and CleanQ30 of 92.80– 94.50 (**Supplementary Table S1**). This indicated that the obtained RNA-Seq data were of high quality and suitable for further analysis. The transcriptome sequencing data were matched with the HFTH1 apple reference genome with 94.76%–97.19% similarity for the HFTH1 and 93.75%–95.19% similarity for the HF. The high matching rate ensured the utilization and accuracy of the transcriptome data.

**Figure 2:**
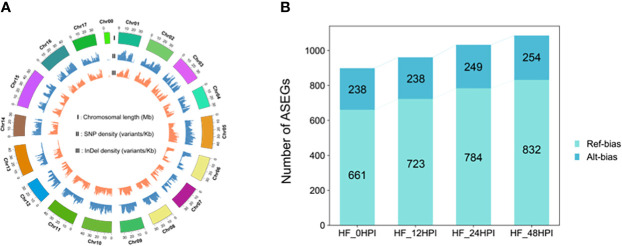
Identification of ASEGs. **(A)** Circos plot of HF resequencing data, chromosome length, SNP density, and indel density. **(B)** The number of ASEGs in HF leaves at 12, 24, and 48 h after AAAP inoculation and mock inoculation (0 HPI).

Whole genome resequencing of heterozygous diploid HF was performed at a sequencing depth of 35×, and a total of 571,019 SNPs and 695,491 indels (**Figure 2A**) were identified using the HFTH1 genome as a reference. To improve the accuracy of ASE analysis, 52,765–46,854 SNP sites (**Supplementary Table S2**) located in the CDS region of genes and shared in both genome and transcriptome with transcriptome sequencing depths > 8 were retained in combination with transcriptome data of HF at each period post AAAP inoculation and control (Gao et al., 2021). These SNPs were used to analyze the expression levels between alleles. Alleles located in the HFTH1 reference genome with expression ratios > 0.75 or < 0.25 were considered to have ASE, whereas the presence of ASE in at least two of the three replicate samples with the same orientation was identified as an ASE gene (ASEG). Finally, a total of 2406 ASEGs were identified in the transcriptome, and 899–1086 ASEGs were identified in each of the four time periods (**Figure 2B**). Most ASEGs were biased toward reference genome expression during at each time period after AAAP inoculation and mock inoculation, and more genes exhibited ASE as the time of AAAP infection progressed (**Supplementary Table S3**). These results suggested that AAAP infection may have altered the allele expression pattern in HF leaves.

### 3.3 Two modes of ASEGs at different time periods before and after AAAP infection

**Figure 3:**
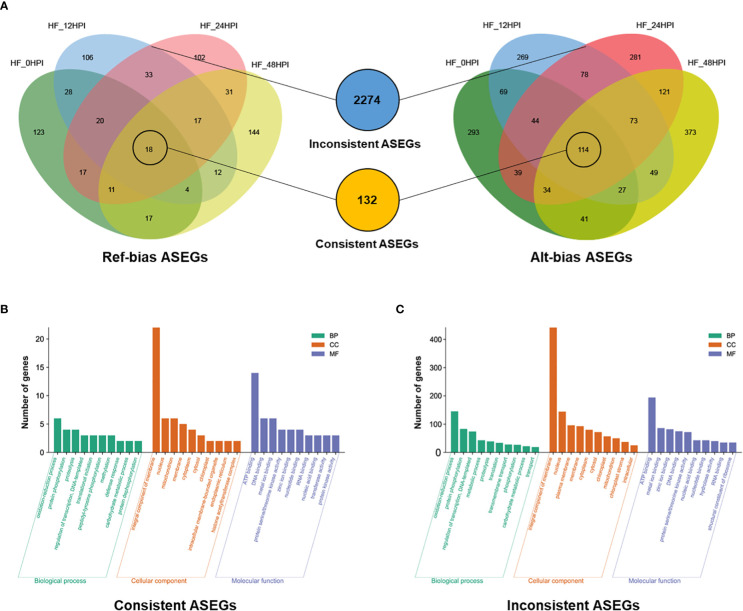
Characteristics of the two models of ASEGs. **(A)** Venn diagram analysis of reference genomic bias (Ref-bias) and alternative genomic bias (Alt-bias) at various times before and after AAAP infection. The middle blue and orange circles indicate inconsistent and consistent ASEGs, respectively. GO enrichment analysis of **(B)** consistent and **(C)** inconsistent ASEGs.

A comparative analysis of ASEGs at different time periods before and after AAAP infection revealed that 132 genes exhibited ASE with the same direction of expression bias before and after the infection. Of them, 114 genes were biased toward the reference genome expression and 18 genes were biased toward the alternative genome expression (**Figure 3A**). Several studies have suggested that genes that exhibit consistent ASE across conditions may have dominant effects (Shao et al., 2019). GO enrichment analysis was used to analyze the biological functions of these ASEGs (**Supplementary Table S4**), and 89 genes were grouped into three main categories of GO: biological process, cellular component, and molecular function (**Figure 3B**). In the category of biological processes, more genes were involved in “oxidation-reduction process,” “protein phosphorylation,” and “proteolysis.” In the category of cellular components, most genes were classified as “integral component of membrane.” In the category of molecular functions, most genes were involved in “ATP binding.”

The remaining 2274 genes of the 2406 ASEGs exhibited inconsistent patterns of allelic bias expression at different time periods before and after the infection (**Figure 3A**). The expression pattern of this allele may cause an overdominance effect in heterozygotes (Zhang et al., 2021). GO enrichment analysis of these genes (**Supplementary Table S5**) revealed that in the category of biological processes, most of the genes were involved in “oxidation-reduction process,” “protein phosphorylation,” and “regulation of transcription, DNA-templates” (**Figure 3C**). In the category of cellular components, the vast majority of genes were classified as “integral component of membrane.” Among the category of molecular functions, most of the genes were enriched in “ATP binding,” “metal ion binding,” and “zinc ion binding.” These results were similar with the enrichment results of biased consistent ASEGs, suggesting that ASEGs may function mainly in some specific subcategories of cellular components and biological processes. These biological processes are closely related to plants’ resistance to pathogens (Turra et al., 2014; David et al., 2019), predicting that ASEGs may play an important role in the process of plant immune response.

### 3.4 Differential expression analysis and KEGG functional annotation

**Figure 4:**
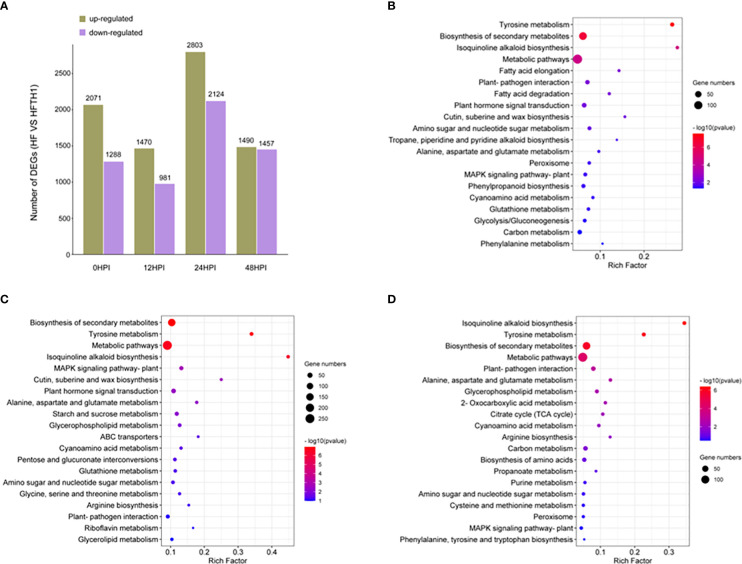
Comparative transcriptomic analysis of HFTH1 and HF before and after AAAP infection. **(A)** DEGs in HF compared with HFTH1 at the same time before and after AAAP infection. **(B–D)** Compared with HFTH1, HF up-regulated the KEGG pathway with significant enrichment of DEGs at 12 HPI **(B)**, 24 HPI, and **(C)** and 48 HPI **(D)**.

To identify defense genes that affect the difference in sensitivity of HF and HFTH1 to AAAP, we performed a comparative analysis of the transcriptomic data of HF and HFTH1 at 12, 24, and 48 h after the infection and mock inoculation (control; 0 HPI). Gene expression was compared between sample sets at different time periods after AAAP infection and mock inoculation, and differentially expressed genes (DEGs) that were up- and downregulated were counted. The results revealed that the up- and downregulated DEGs after AAAP infection in HF and HFTH1 increased with the advancement of infection time, compared with the control (**Supplementary Figures S1A-D**). This suggested that more genes were involved in the response to AAAP as the time of infection advanced. Meanwhile, the number of DEGs, both up- and downregulated, was higher in HF than in HFTH1 at each post-infection time compared with the control (**Supplementary Figures S1A, B**). This indicated that more genes were involved in disease-resistant varieties in response to AAAP stress. The transcriptome data of HF and HFTH1 in the same time after infection and mock inoculation were analyzed, and the DEGs that were up- and downregulated by HF compared with HFTH1 at each time period were separately counted. Due to the similar genetic background between the two species, relatively few DEGs were obtained. Moreover, and the number of DEGs upregulated in HF relative to HFTH1 was higher than that of DEGs downregulated in all four time periods after infection and mock inoculation (**Figure 4A**).

Based on orthology (KO) terminology mapping of the upregulated DEGs of HF compared with HFTH1 at the same period after pathogenic inoculation to the KEGG pathway, the top 20 significantly enriched pathways at each time period were selected for analysis (**Figures 4B-D**). Multiple KEGG pathways were significantly simultaneously enriched in the comparative combinations at each time period; they were “Tyrosine metabolism,” “Biosynthesis of secondary metabolites,” “Isoquinoline alkaloid biosynthesis,” “Metabolic pathways,” “Plant-pathogen interaction,” “Amino sugar and nucleotide sugar metabolism,” “Alanine, aspartate, and glutamate metabolism,” “MAPK signaling pathway-plant,” and “Cyanoamino acid metabolism.” Among them, “Plant-pathogen interaction” and “MAPK signaling pathway-plant” are directly related to the defense response of plants against pathogens (Dodds and Rathjen, 2010). MAPK signaling is a central pathway in immune response transduction and plays a crucial role in phytohormone-induced immune responses (Meng and Zhang, 2013). DEGs enriched in “Plant-pathogen interaction” and “MAPK signaling pathway-plant” at three time periods after pathogenic infection comprised a dataset containing 51 “core” immune genes (**Supplementary Table S6**). This dataset provides candidate genes for further analysis and functional identification.

### 3.5 Identification of *MdFLS2* and expression analysis of related genes

**Figure 5:**
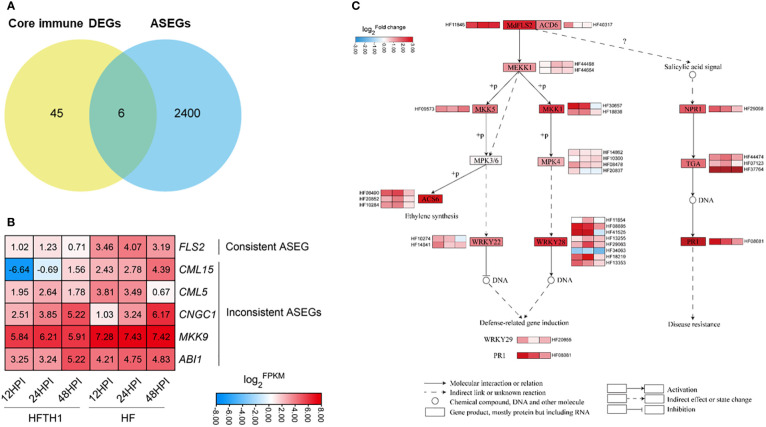
The ASE gene *MdFLS2* may affect the defense response of apple leaves to AAAP. **(A)** Venn diagram analysis of “core” immune DEGs and ASEGs. **(B)** Heatmap showing log_2_^FPKM^ of six “core” immune ASEGs in HFTH1 and HF at 12, 24, and 48 h after AAAP inoculation. **(C)** Heatmap sequentially shows the log_2_^Fold change^ of genes from *MdFLS2*-related pathway in HF compared with HFTH1 at 12, 24, and 48 h after AAAP inoculation.

Combining the above “core” immune genes dataset with the 2406 ASEGs, the Venn diagram indicated that six genes were simultaneously present in both datasets (**Figure 5A**). Heatmaps of the expression of these genes between HF and HFTH1 leaves indicated that *MdFLS2* consistently exhibited significant differences in expression levels at three time periods after AAAP infection (**Figure 5B**). Meanwhile, *MdFLS2* was consistently biased toward alternative genomic ASE after AAAP infection and mock inoculation. Previous studies have reported that overexpression of *MdFLS2* in *Arabidopsis* enhances salicylic acid signaling and improves resistance to *Botryosphaeria dothidea* in *Arabidopsis* (Liu et al., 2018). There are some similarities in the disease resistance mechanisms of apples to AAAP and *Botryosphaeria dothidea*; for example, sorbitol can enhance the resistance of apples to both AAAP and *Botryosphaeria dothidea* (Meng et al., 2018; He et al., 2022).

To further clarify the differential expression of genes from *MdFLS2*-related pathway between the two plant materials, we analyzed the differential expression of related pathway genes in HFTH1 and HF, including those from “Plant-pathogen interaction,” “MAPK signaling pathway-plant,” and salicylic acid pathway in plant hormone signal transduction. Heatmap analysis revealed that the expression levels of *MdFLS2*-related pathway genes were basically higher in HF than in HFTH1; particularly, expression levels of genes related to salicylic acid pathway were significantly higher in disease-resistant HF than disease-susceptible HFTH1 at the early stage of pathogenic infection, i.e., at 12 HPI and 24 HPI after AAAP inoculation (**Figure 5C**). These genes may play an essential role in the early defense against pathogenic infection.

### 3.6 *MdFLS2-1* is a positive regulator of resistance to AAAP

The *MdFLS2* allele located in the HF alternative genome was named *MdFLS2-1*, and that located in HFTH1 was named *MdFLS2-2*. ASE analysis revealed that *MdFLS2-1* exhibited a significant expression advantage compared with *MdFLS2-2* in HF after AAAP infection and mock inoculation. To identify the allelic variants located in the CDS region of the *MdFLS2*, we cloned *MdFLS2-1* and *MdFLS2-2* in HF leaves. *MdFLS2-1* and *MdFLS2-2* had 10 SNPs in the CDS region and caused difference in five amino acids. By comparing the protein sequence of FLS2 with that of *A. thaliana* (Gomez-Gomez and Boller, 2000), *Citrus sinensis*, *Prunus dulcis*, *Rosa chinensis*, *Trifolium pratense*, *Medicago truncatula*, *Glycine max*, and *P. persica*, we discovered that the amino acid sites of the variants were not conserved in several species (**Supplementary Figure S2**). However, these results are insufficient to indicate whether amino acid variants of MdFLS2-1 and MdFLS2-2 cause functional differences.

**Figure 6:**
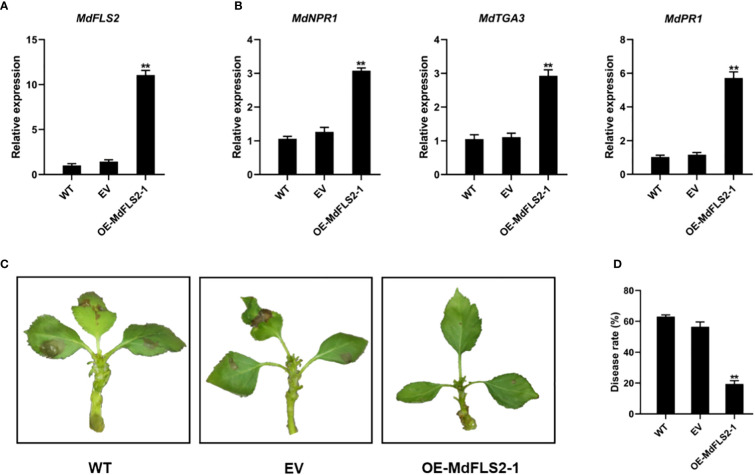
MdFLS2-1 positively regulates resistance of apple to AAAP. RT-qPCR analysis of the expression level of **(A)** MdFLS2 and **(B)** salicylate pathway marker genes MdNPR1, MdTGA3, and MdPR1 in leaves of WT, EV, and OE-MdFLS2-1 plants. Asterisks denote Student’s t-tests, significance: **P < 0.01. The mean ± SD of biological triplicates were taken for every value. **(C)** Symptoms of infection in leaves of WT, EV, and OE-MdFLS2-1 plants at 48 h after AAAP inoculation. **(D)** Incidence statistics of leaves of WT, EV, and OE-MdFLS2-1 plants at 48 h after AAAP inoculation. Values are the means ± SD of three biological replicates (Student’s t-tests, **P < 0.01).

We observed that the previously reported *MdFLS2* is actually *MdFLS2-2* (Liu et al., 2018). To determine the function of *MdFLS2-1* and its role in AAAP infection, we constructed the PRI01-*MdFLS2-1* vector and performed transient expression experiments in GL-3 apple leaves. RT-qPCR analysis of transgenic leaves revealed that *MdFLS2* expression levels were significantly increased in OE-MdFLS2-1 transgenic leaves compared with those in untransformed control leaves (WT) with empty vector (EV) (**Figure 6A**). AAAP inoculation tests on transgenic leaves and controls revealed that apple leaves overexpressing *MdFLS2-1* had significantly less disease incidence than WT and EV plants at 48 HPI (**Figures 6C, D**). These results suggested that *MdFLS2-1* positively regulates resistance to AAAP in apple. RT-qPCR analysis of genes related to salicylic acid pathway in transgenic leaves revealed that the expression levels of marker genes of salicylic acid signaling pathway (*MdNPR1*, *MdTGA3*, and *MdPR1*) were significantly higher in OE-MdFLS2-1 plants compared with WT and EV plants (**Figure 6B**). These results suggested that the expression of both *MdFLS2-1* and *MdFLS2-2* is positively correlated with the salicylic acid signaling pathway, and *MdFLS2-2* positively regulates the resistance of apple leaves to AAAP. Therefore, we speculated that *MdFLS2-1* and *MdFLS2-2* play at least similar positive roles in the resistance of apples to fungi.

### 3.7 TRIM-like sequence as an enhancer of *MdFLS2-1* expression

**Figure 7:**
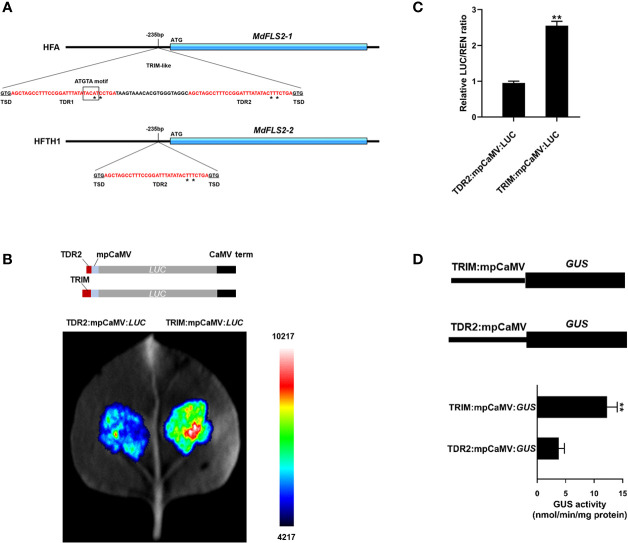
Insertion of the TRIM-like sequence affects the expression level of *MdFLS2* allele. **(A)** Molecular structure of *MdFLS2-1* from HF alternative genome (HFA) and of *MdFLS2-2* from HFTH1 reference genome with flanking sequence. The TRIM-like sequence contains the following sequence features: TSD (black underlined), TDRs (red font), and intermediate sequence (black font). Base differences between TDR1 and TDR2 are marked with asterisks. The black box indicates the ATGTA motif. **(B)** Transient expression analysis revealed that TRIM-like sequence insertion could significantly increase the expression level of luciferase. Upper panel represents the TDR2:mpCaMV:*LUC* (up) and TRIM:mpCaMV:*LUC* (down) constructs, both containing the minimal promoter from the cauliflower mosaic virus (mpCaMV), luciferase ORF, and cauliflower mosaic virus terminator (CaMV term), to which the TDR2 sequence and TRIM-like sequence are respectively added. Lower panel indicates luciferase assay of Nicotiana benthamiana leaves 72 h after infection with Agrobacterium strains containing TDR2:mpCaMV:LUC (left) and TRIM:mpCaMV:*LUC* (right). **(C)** The relative ratio of LUC/REN for both TDR2:mpCaMV:*LUC* and TRIM:mpCaMV:*LUC*. Values are the means ± SD of three biological replicates (Student’s t-tests, **P < 0.01). **(D)** GUS activity in the transiently transformed N. benthamiana leaves with constructs TRIM:mpCaMV:GUS and TDR2:mpCaMV:GUS under normal conditions. The mean ± SD of biological triplicates were taken for every value. Asterisks denote significant differences by Student’s t-test (**P < 0.01).

Since in heterozygous diploids, alleles are placed in the same *trans* regulation background and cellular environment, the effect of *trans* regulation on allele expression can be temporarily shielded, thus prioritizing *cis* regulation of variants in the genome (Hill et al., 2021). To investigate the *cis*-regulatory mechanism causing specific expression of the *MdFLS2* allele, we searched the promoter region of the *MdFLS2* allele using whole-genome resequencing data of HF. Based on resequencing data and *MdFLS2* allele promoter cloning, we identified an 85-bp TRIM-like sequence insertion in the promoter of *MdFLS2-1*, which was located 235-bp upstream of the translation start codon (**Figure 7A**). The TRIM-like sequence consists of two almost identical 32-bp TDRs and a 21-bp intermediate sequence (**Figure 7A**). The TRIM-like sequence is flanked by 3-bp target site duplication that was generated after insertion. The two TDRs of the TRIM-like element have two base differences, named TDR1 and TDR2, respectively. In the promoter region of *MdFLS2-2*, the TRIM-like sequence undergoes truncation, retaining only a TDR2 sequence (**Figure 7A**).

Several studies have reported that transposon insertion in the promoter region can provide enhancer activity and thus increase the expression level of genes (Wei and Cao, 2016). *MdFLS2* alleles with intact transposon insertion in the promoter region had higher expression levels, which is consistent with the pattern of transposon insertions enhancing gene expression. To determine whether the insertion of the TRIM-like element provided enhancer activity, we ligated an 85-bp TRIM-like sequence inserted in the promoter of *MdFLS2-1* to the 35S minimal promoter and performed transient experiments in leaves of *N. benthamiana* to assess the effect of the TRIM-like element on the expression of firefly luciferase gene and β-glucuronidase gene. The results revealed that the TRIM-like sequence led to a significant increase in reporter gene expression, relative to the use of only a TDR2 sequence from the promoter of *MdFLS2-2*-containing structure (**Figures 7B-D**). This suggested that the insertion of the TRIM-like sequence in the promoter region of *MdFLS2-1* increased the expression level of the gene, causing differential expression of the *MdFLS2* allele in HF leaves. This natural variation in the promoter region of the *MdFLS2* allele resulted in its significantly higher expression level in HF than in the homozygote HFTH1.

## 4 Discussion

ASE analysis in heterozygous organisms provides an unprecedented method to compare the effects of *cis*-regulatory variants on gene expression in the same *trans*-regulatory background and cellular environment (Bao et al., 2019). Genome-wide ASE analysis was performed by RNA-seq of leaves of HF, an important disease-resistant cultivar, after AAAP infection and mock infection. A large number of ASE genes were identified. These genes form a validated dataset to rapidly identify the *cis*-regulatory mechanisms affecting the expression levels of these alleles by comparing genomes.

### 4.1 *MdFLS2* is involved in the resistance of apple leaves against AAAP

PTI is an important mode of plant immunity (Bigeard et al., 2015). Some receptor kinases are involved in the resistance of plants to fungi by linking hormone signals (Tang et al., 2017). By comparative transcriptomic analysis, we identified an important receptor kinase, *MdFLS2*, that exhibited significant differences in expression between HF and HFTH1 after AAAP inoculation. There was a consistent bias of ASE for *MdFLS2* in HF after AAAP infection and mock inoculation, and the expression level of the HF-derived allele *MdFLS2-1* was significantly higher than that of the HFTH1-derived allele *MdFLS2-2*. Previous studies have reported that *MdFLS2-2* is involved in the defense response of apple to fungi by enhancing the expression levels of the genes related to salicylic acid pathway. In the transcriptome data, we observed an association of *MdFLS2* with the genes related to salicylic acid pathway, with their higher expression levels in the AAAP-resistant HF compared with AAAP-susceptible HFTH1 at the early stage of AAAP infection; the genes down-regulated at a later stage. Related studies have indicated that an intact salicylic acid pathway is essential in the defense of *Solanum tuberosum* against *Alternaria solani* (Brouwer et al., 2020). Studies on the defense of *Chrysanthemum morifolium* against *Alternaria* (Liu et al., 2020; Zhao et al., 2020) have reported that the salicylic acid pathway plays an important role in the early response of plants against pathogenic attacks. Transient overexpression of *MdFLS2-1* could improve resistance of apple leaves to AAAP (**Figures 6C, D**). At the same time, we observed that overexpression of *MdFLS2-1* was accompanied by increased expression levels of genes related to salicylic acid pathway (**Figures 6A, B**). These results predicted a conserved and active role of salicylic acid pathway in the defense of various plants against various pathogenic variants of *Alternaria*. On the other hand, *MdFLS2-1* and *MdFLS2-2* could regulate the level of salicylic acid signaling in apple leaves during resistance to fungi.

Studies on *Arabidopsis* indicated that AtFLS2 receptor kinase can function in complex with various proteins, including AtACD6, which can form a complex with AtFLS2 to enhance salicylic acid signaling (Tateda et al., 2014), and AtACD6 and AtFLS2 can promote the activation of salicylic acid signaling even in the absence of PAMPs (Tateda et al., 2015). Significant differences in *AtACD6* homolog *MdACD6* were reflected at 12 HPI in susceptible varieties, which is consistent with the expression trend of genes related to salicylic acid pathway (**Figure 5C**). We speculated that a similar mechanism may exist in apple, where *MdFLS2* regulates salicylic acid signaling through *MdACD6*. However, further studies are needed to elucidate this mechanism.

### 4.2 A TRIM-like insertion in *MdFLS2* allele promoter causes its ASE

**Figure 8:**
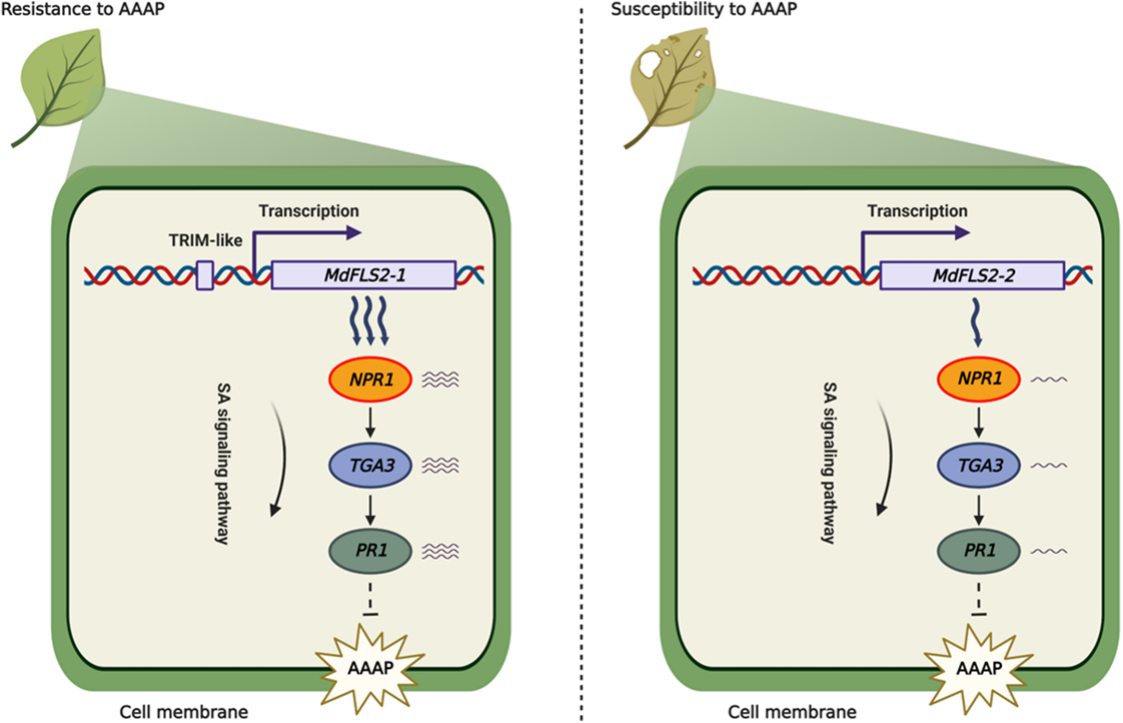
Model for the role of a TRIM-like sequence insertion in the *MdFLS2-1* promoter region in regulating resistance of apple to Alternaria blotch disease. *MdFLS2* allele is involved in the resistance of apple to Alternaria blotch disease *via* increasing the expression level of genes related to salicylic acid pathway (*NPR1*, *TGA3*, and *PR1*). Insertion of a TRIM-like sequence into the *MdFLS2-1* promoter region enhances *MdFLS2-1* expression to improve resistance of apple to Alternaria blotch disease. Arrow: activation; Bar: repression.

It is widely reported that insertion of transposable elements in the promoter region affects expression level of the genes (Mao et al., 2015; Xia et al., 2017; Niu et al., 2022). In this study, we identified a TRIM-like sequence inserted in the promoter region of *MdFLS2-1*, which was experimentally demonstrated to have enhancer activity. In contrast, promoter region of the other allele *MdFLS2-2* only had a TDR2 sequence of the TRIM-like element; thus, the expression level of *MdFLS2-2* was significantly lower than that of *MdFLS2-1*. Based on these results, we developed a model to explain the difference in the sensitivity of HF and HFTH1 to AAAP. After AAAP infection, HF containing *MdFLS2-1* exhibited high expression levels of genes related to salicylic acid pathway, which in turn led to resistance of HF to AAAP. In contrast, HFTH1 with lower expression levels of *MdFLS2-2* exhibited susceptibility to AAAP (**Figure 8**).

Some studies have reported that insertion of transposable elements can provide effective binding sites for transcription factors (Sahebi et al., 2018). An ATGTA motif is present in the TDR1 sequence of the TRIM-like element. Due to mutations during evolution, the ATGTA motif is not present in the TDR2 sequence. Previous studies have identified the ATGTA motif as a binding site for the *MdEIL1* transcription factor in apple (An et al., 2018). Interestingly, *AtEIL1*, the homolog of *MdEIL1* in *Arabidopsis*, can bind to the promoter region of *AtFLS2* to regulate its expression level (Boutrot et al., 2010). In apples, this regulatory mechanism is expected to be further revealed in future studies. Overall, our study provided new insights into the molecular mechanism of resistance to AAAP by apple and provided a reference for breeding with disease resistance varieties.

## Data availability statement

The datasets presented in this study can be found in online repositories. The names of the repository/repositories and accession number(s) can be found below: National Center for Biotechnology Information (NCBI) BioProject database under BioProject ID: PRJNA896728 and PRJNA897033.

## Author contributions

LZ, PC and ZL initiated the project and designed the study. ZL, KL, CJ, AY, JY performed biological experiments. ZL performed data analysis and wrote the manuscript. XH, CZ optimized experimental protocol. LZ, and PC revised the manuscript. All authors contributed to the article and approved the submitted version.
