# Supplementary material for: Insertion of a TRIM-like sequence in MdFLS2-1 promoter is associated with its allele-specific expression in response to Alternaria alternata in apple
Source: Front Plant Sci. 2022 Dec 29;13:1090621. doi: 10.3389/fpls.2022.1090621 (PMC9834810; doi:10.3389/fpls.2022.1090621)
Supplement: Supplementary file 1 [file DataSheet_1.docx]

Supplementary Material

##
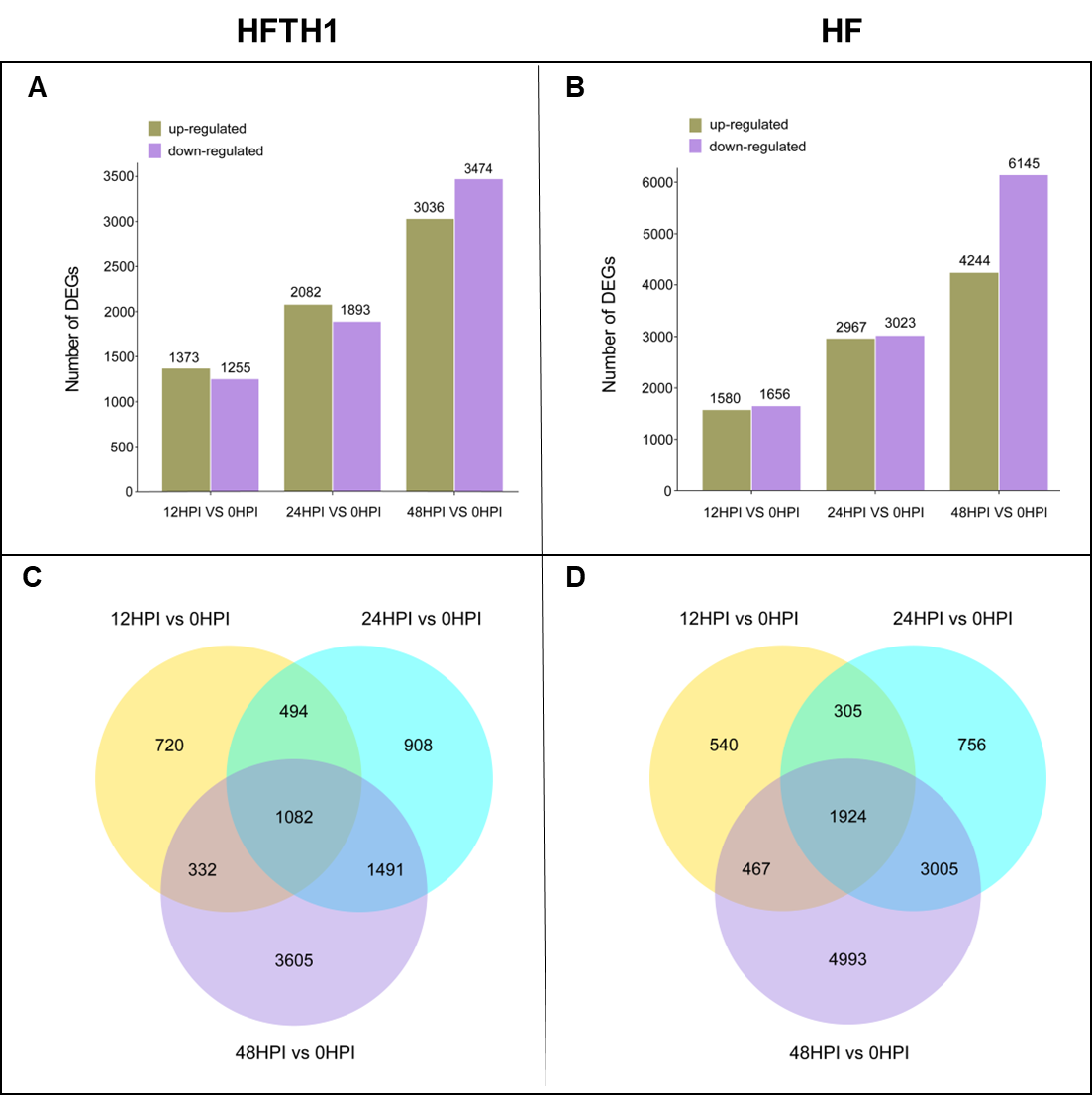


## Supplementary Figure S1. DEGs between AAAP inoculation and mock inoculation. (A-B) DEGs at different times after AAAP inoculation and between mock inoculation. HFTH1 (A); HF (B). (C-D) Venn diagram analysis of DEGs at different times after AAAP inoculation and between mock inoculation. HFTH1 (C); HF (D).


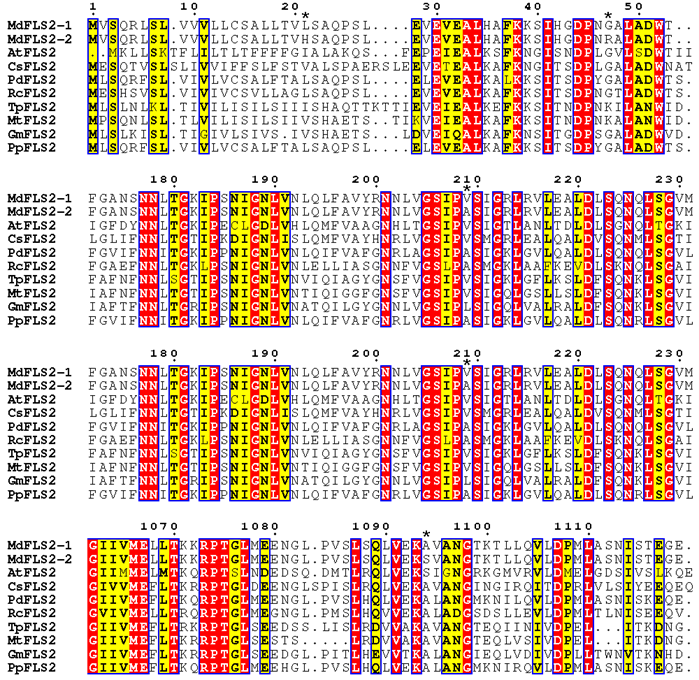


**Supplementary Figure S2.** **Protein sequence alignment of FLS2 from different species.** Red and yellow backgrounds indicate relatively conservative amino acid sites, and asterisks indicate the amino acid sites with differences between MdFLS2-1 and MdFLS2-2.
